# Subsurface microbiology and biogeochemistry of a deep, cold-water carbonate mound from the Porcupine Seabight (IODP Expedition 307)

**DOI:** 10.1111/j.1462-2920.2008.01759.x

**Published:** 2009-01

**Authors:** Gordon Webster, Anna Blazejak, Barry A Cragg, Axel Schippers, Henrik Sass, Joachim Rinna, Xiaohong Tang, Falko Mathes, Timothy G Ferdelman, John C Fry, Andrew J Weightman, R John Parkes

**Affiliations:** 1 Cardiff School of BiosciencesMain Building, Park Place, Cardiff, Wales, UK; 2 School of Earth and Ocean Sciences, Cardiff UniversityMain Building, Park Place, Cardiff, Wales, UK; 3 Federal Institute for Geosciences and Natural Resources, Section GeomicrobiologyStilleweg 2, 30655 Hannover, Germany; 4 Max Planck Institute for Marine MicrobiologyCelsiusstr. 1, D-28359 Bremen, Germany

## Abstract

The Porcupine Seabight Challenger Mound is the first carbonate mound to be drilled (∼270 m) and analyzed in detail microbiologically and biogeochemically. Two mound sites and a non-mound Reference site were analyzed with a range of molecular techniques [catalyzed reporter deposition-fluorescence *in situ* hybridization (CARD-FISH), quantitative PCR (16S rRNA and functional genes, *dsrA* and *mcrA*), and 16S rRNA gene PCR-DGGE] to assess prokaryotic diversity, and this was compared with the distribution of total and culturable cell counts, radiotracer activity measurements and geochemistry. There was a significant and active prokaryotic community both within and beneath the carbonate mound. Although total cell numbers at certain depths were lower than the global average for other subseafloor sediments and prokaryotic activities were relatively low (iron and sulfate reduction, acetate oxidation, methanogenesis) they were significantly enhanced compared with the Reference site. In addition, there was some stimulation of prokaryotic activity in the deepest sediments (Miocene, > 10 Ma) including potential for anaerobic oxidation of methane activity below the mound base. Both *Bacteria* and *Archaea* were present, with neither dominant, and these were related to sequences commonly found in other subseafloor sediments. With an estimate of some 1600 mounds in the Porcupine Basin alone, carbonate mounds may represent a significant prokaryotic subseafloor habitat.

## Introduction

In recent years, large clusters of giant carbonate mud mounds, some more than 300 m high, have been discovered off the continental margins of Europe (Wheeler *et al*., 2007). They are accumulations that generally occur in localized clusters and vary in size and shape being conical-, ridge- and ring-shaped, and in some cases having very steep sides. Large and small dome-shaped knolls which lie on the surface of the sea-floor have been described, as well as complex knolls and pinnacle knolls (Hovland *et al*., 1994). The mounds in the Porcupine Seabight are in water depths of 600–900 m and form impressive conical shapes up to 2 km wide and 350 m high (De Mol *et al*., 2002; Kenyon *et al*., 2003). Seismic profiles have shown that some mounds are covered by tens of metres of sediment or dead coral rubble (De Mol *et al*., 2002; Huvenne *et al*., 2007) and others are slowly being buried (e.g. Challenger Mound, Ferdelman *et al*., 2006). There are also living mounds with a thriving community of cold-water corals (*Lophelia pertusa* and *Madrepora oculata*) in close association with other organisms as part of a diverse cold-water coral reef ecosystem (De Mol *et al*., 2007).

However, there is some debate over how these carbonate mounds are initiated and it may be that a variety of mechanisms are operating. In some areas, the origin of these mounds has been related to the seepage of light hydrocarbon and nutrient-rich pore waters through the seafloor initiating microbially induced carbonate formation (Hovland *et al*., 1994) and indirectly stimulating and providing a platform for cold-water coral growth (internal control theory). A second hypothesis is that oceanographic and palaeoenvironmental conditions control mound initiation and growth (external control theory) allowing favourable conditions for colonization by cold-water corals (Wheeler *et al*., 2007).

During the Integrated Ocean Drilling Program (IODP) Expedition 307 (Ferdelman *et al*., 2006) the first complete section through a modern cold-water coral mound, and to beneath its base, was recovered from the Challenger Mound in the Belgica Mound Province of the Porcupine Seabight. The objectives of Expedition 307 were to test whether: (i) gas seeps acted as a prime trigger for mound genesis, (ii) prominent erosional surfaces reflected global oceanographic events, (iii) the mound is a high-resolution palaeoenvironmental recorder, and (iv) these mounds are present-day analogues for Phanerozoic reef and mud mounds.

This study used samples from IODP Expedition 307 to determine the prokaryotic community, its activity and diversity, in a partially buried carbonate mound, using a range of culture and culture-independent methods coupled with biogeochemical depth profiles, in order to assess whether such mounds are still biogeochemically active and thus represent an important subseafloor prokaryotic habitat. Two mound sites, Flank (IODP site U1316) and Mound (IODP site U1317), were compared with a non-mound Reference site (IODP site U1318) upslope from the Challenger Mound.

## Results

### Site description

**Fig. 1:**
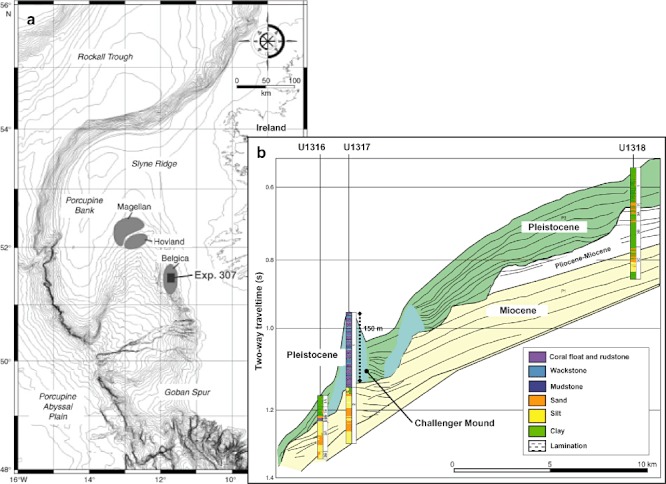
a. Location of IODP Expedition 307 operations area in the Belgica Mound Province, Porcupine Seabight. b. Lithostratigraphy of three drilling sites (U1316, U1317 and U1318) projected on the seismic profile of Challenger Mound along a north-north-west to south-south-east transect.

The Challenger Mound (781–815 m water depth) is a prominent mound structure, 155 m in height and partially buried with sediment and dead cold-water coral (*Lophelia pertusa*) rubble (Ferdelman *et al*., 2006). It is one of 66 mounds which make up the Belgica Mound Province in the Porcupine Seabight on the south-west Irish continental margin (Ferdelman *et al*., 2006; Fig. 1). In summary, drilling revealed that Challenger Mound rests on a sharp erosional boundary with sediments below consisting of glauconitic and silty-sandstone of early middle Miocene age. The erosional feature can be attributed to an Atlantic basin-wide erosional event that deeply cut into the middle Miocene strata at the mound sites U1316 and U1317 (van Rooij *et al*., 2003; Louwye *et al*., 2008). This erosional unconformity also appears in Reference site U1318 sediments (∼84 m below seafloor; mbsf), separating Miocene sediments of 10 Ma from Middle Pleistocene or younger sediments (< 0.7 Ma; Kano *et al*., 2007; Louwye *et al*., 2008). At the Challenger Mound site (U1317), the Miocene stratum ends in firmground that is overlain by late Pliocene-Pleistocene mound succession consisting of floatstone and rudstone formed of fine sediments and cold-water branching corals. Strontium isotope stratigraphy on coral pieces suggests that growth of Challenger Mound began at 2.6 Ma and rapidly grew at rates up to 24 cm ka^−1^ until 1.7 Ma (Kano *et al*., 2007). After a hiatus of 0.7 Ma mound growth resumed until 0.5 Ma. Recent sediments of < 500 ka are absent and the mound has little or no live cold-water coral. Downslope of the Challenger Mound the Flank site (Fig. 1, U1316) consists of a 10- to 13-m-thick wedge of coral bearing sediment between 45.2 and 58.3 mbsf. Fifty metres of late Pleistocene-Recent silty-clays, frequently containing dropstones, cover the coral-bearing layer (Ferdelman *et al*., 2006). The upslope Reference site (Fig. 1, U1318, 423 m water depth) consists of three main lithostratigraphic units. The overlying sediment down to 82 mbsf consists of rapidly depositing silt-clays less than 260 ka. A middle section of 2 m thickness consists of conglomerates and pebbles, whereas the deeper Miocene unit consists of silty-clays. The upper Miocene section that is missing at the downslope Mound and Flank sites is present at this Reference site U1318 and is approximately 40 m thick (86–127 mbsf). Palynological and palaeomagnetic data suggest that this sequence is < 3.6 Ma old (Louwye *et al*., 2008).

### Contamination checks

Perfluorocarbon tracer (PFT) was injected continuously into the drilling fluid during coring of all sites (1 ml PFT l^−1^ seawater drill fluid). The PFT tracer was not detected in any inner core samples (Ferdelman *et al*., 2006) used for microbiological analysis. Fluorescent microspheres were also deployed in all cores from which microbiological samples were taken. Microspheres in samples taken midway between the core middle and the outside of the core were detected in three samples at site U1316 (17–42 microspheres cm^−3^ sediment), six samples at site U1317 (21–993 microspheres cm^−3^ sediment) and five samples at site U1318 (16–2425 microspheres cm^−3^ sediment). Although it is not possible to accurately quantify the level of possible microbial contamination within a sample, it should be noted that if these numbers are compared with the original microsphere suspension of 7 × 10^6^ microspheres μl^−1^ then the highest number of microspheres detected at Flank site U1316 (9.95–9.90 mamb, metres above mound base), Mound site U1317 (109.25–109.20 mamb) and Reference site U1318 (23.55–23.60 mbsf) is equivalent to 0.006, 0.14 and 0.35 nl of seawater drilling fluid per cm^−3^ sediment, which is much less than 1 cell cm^−3^ taking seawater prokaryotic cell numbers to be 4.2 × 10^8^ l^−1^ (Ferdelman *et al*., 2006).

### Prokaryotic cell numbers

#### Acridine orange direct counts

**Fig. 2:**
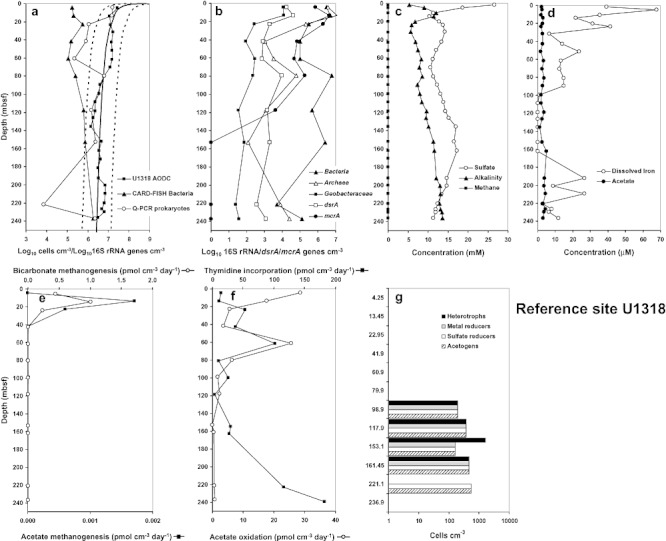
Depth profiles of prokaryotic cell numbers, prokaryotic activity and geochemical data for Reference site U1318. a. Prokaryotic cell numbers determined by AODC and qPCR of 16S rRNA genes, and bacterial CARD-FISH. The solid line shows Parkes and colleagues (2000) general model for prokaryotic cell distributions in deep marine sediments, and dotted lines represent 95% prediction limits. b. DNA copy numbers of the 16S rRNA genes determined by qPCR of *Bacteria*, *Archaea* and *Geobacteraceae*, *mcrA* and *dsrA* genes. c and d. Pore water concentrations of sulfate, alkalinity, dissolved Fe and acetate, and *in situ* methane. e. Potential rates of methanogenesis from H_2_ : CO_2_ and acetate. f. Rates of thymidine incorporation and acetate oxidation to CO_2_. g. Culturable cells from MPN enrichments; heterotrophic, metal-reducing, sulfate-reducing and acetogenic bacteria.

At the Reference site (U1318, Fig. 2) the depth profile of total acridine orange direct count (AODC) cell numbers generally followed the global trend observed in deep subseafloor sediments at other Ocean Drilling Program (ODP) sites (Parkes et al., 2000). At the near sediment surface (4.85 mbsf) total cell numbers were 2.7 × 10^7^ cells cm^−3^ decreasing twofold by 14.05 mbsf and then remained relatively constant down to 70.47 mbsf (Fig. 2A), suggesting maintenance of an active prokaryotic population which was supported by a high proportion of dividing cells (average to 80 mbsf = 9%, n = 9), calculated as a percentage of the AODC. Across the ∼84 mbsf erosional surface there was a threefold decrease in total prokaryotic cell numbers down to 89.5 mbsf, from where prokaryote populations decreased at a much greater rate than the global average regression line down to 135.5 mbsf. Also within this depth range, few or no dividing cells were observed (Ferdelman et al., 2006), suggesting that populations were in decline and/or stressed. Interestingly, below ∼140 mbsf cell numbers then recovered and followed closely the global average until 210 mbsf where counts become elevated to 7 × 10^6^ cells cm^−3^ (Fig. 2A).

**Fig. 3:**
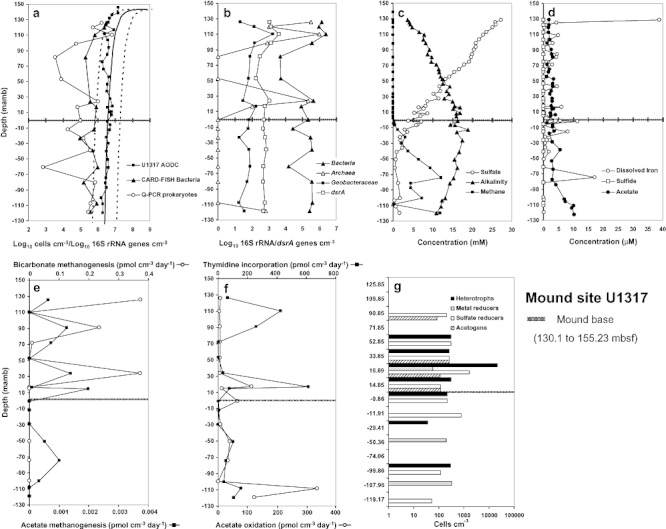
Depth profiles of prokaryotic cell numbers, prokaryotic activity and geochemical data for Mound site U1317. a. Prokaryotic cell numbers determined by AODC and qPCR of 16S rRNA genes, and bacterial CARD-FISH. The solid line shows Parkes and colleagues (2000) general model for prokaryotic cell distributions in deep marine sediments, and dotted lines represent 95% prediction limits. b. DNA copy numbers of the 16S rRNA genes determined by qPCR of *Bacteria*, *Archaea* and *Geobacteraceae*, and *dsrA* genes. c and d. Pore water concentrations of sulfate, alkalinity, dissolved Fe, sulfide and acetate, and *in situ* methane. e. Potential rates of methanogenesis from H_2_ : CO_2_ and acetate. f. Rates of thymidine incorporation and acetate oxidation to CO_2_. g. Culturable cells from MPN enrichments; heterotrophic, metal-reducing, sulfate-reducing and acetogenic bacteria.

In contrast to the Reference site the depth profile of cell numbers at the Mound site (U1317) initially (down to ∼35 mbsf; 110 mamb) followed the lower regression limits for global cell numbers (Parkes *et al*., 2000), with a prokaryote population (1.4 × 10^7^ to 2.5 × 10^6^ cells cm^−3^) smaller than expected (Fig. 3A). Between ∼120 and 75 mamb there was a small increase in cell numbers accompanied by an increase in dividing cell numbers (data not shown). After ∼65 mamb, cell numbers further increased, reaching and at some depths (∼30–6.5 mamb) becoming higher than the global average for the remainder of the core (Fig. 3A). Additionally, this increase in subsurface cell numbers was accompanied by a steady rise in dividing cells down to −86.25 mamb which fluctuated between 0% and ∼14%, suggesting a more active population.

**Fig. 4:**
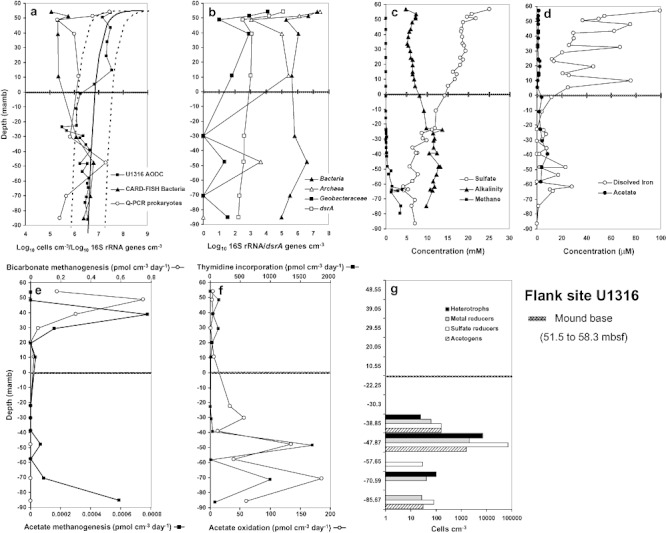
Depth profiles of prokaryotic cell numbers, prokaryotic activity and geochemical data for Flank site U1316. a. Prokaryotic cell numbers determined by AODC and qPCR of 16S rRNA genes, and bacterial CARD-FISH. The solid line shows Parkes and colleagues (2000) general model for prokaryotic cell distributions in deep marine sediments, and dotted lines represent 95% prediction limits. b. DNA copy numbers of the 16S rRNA genes determined by qPCR of *Bacteria*, *Archaea* and *Geobacteraceae*, and *dsrA* genes. c and d. Pore water concentrations of sulfate, alkalinity, dissolved Fe and acetate, and *in situ* methane. e. Potential rates of methanogenesis from H_2_ : CO_2_ and acetate. f. Rates of thymidine incorporation and acetate oxidation to CO_2_. g. Culturable cells from MPN enrichments; heterotrophic, metal-reducing, sulfate-reducing and acetogenic bacteria.

The depth distribution of AODC cell number at the Flank site (U1316) also followed the global average with depth, although there were some substantial deviations (Fig. 4A). Numbers decreased rapidly from 3.1 × 10^7^ cells cm^−3^ at the sediment surface (1.49 mbsf; 53.57 mamb) to 1.8 × 10^6^ cells cm^−3^ at approximately the mound base (56.19 mbsf; −1.13 mamb). Below the mound base, cell numbers declined further, but more slowly, to 3.1 × 10^5^ cells cm^−3^, the smallest population measured at any of the three sites (at −23.63 mamb). Subsequently, cell numbers increased (10-fold) and remained within global regression limits down to −86.25 mamb. The numbers of dividing cells were high (17%) near the sediment surface and then decreased to 0% at the mound base. Below the mound base dividing cell numbers remained at zero until −29.63 mamb where they increased to 12.5%, corresponding with an increase in the prokaryotic population, and from there down to −86.25 mamb the numbers of dividing cells fluctuated between 0% and ∼14% (Ferdelman *et al*., 2006).

#### CARD-FISH and prokaryotic quantitative PCR analysis

At all three sites archaeal catalyzed reporter deposition-fluorescence *in situ* hybridization (CARD-FISH) counts were below the 10^5^ cells cm^−3^ detection limit (Schippers *et al.*, 2005) and therefore CARD-FISH cell counts were only obtained with the bacterial probe mixture EUB338-I to -III. At the Reference site (U1318) cell numbers determined by bacterial CARD-FISH and quantitative PCR (qPCR) of DNA copy numbers of prokaryotic 16S rRNA genes using a universal primer set (Takai and Horikoshi, 2000) were considerably lower than the AODC (Fig. 2A). Bacterial CARD-FISH cell counts were very low in the near surface (2 × 10^5^ cells cm^−3^) and gradually increased with depth, whereas qPCR of prokaryotic 16S rRNA gene copy numbers were higher near the surface (1.6 × 10^7^ cells cm^−3^) and variably decreased with depth, with 60% of measured samples falling within the predicted global AODC regression limits.

At the two mound sites (Figs 3A and 4A) cell numbers determined by bacterial CARD-FISH and qPCR of DNA copy numbers of prokaryotic 16S rRNA genes were also generally lower than AODC cell numbers, particularly above the mound base. Specifically, at the Mound site (U1317) a considerable number of samples (60%) counted by CARD-FISH had values that were on or close to the lower global AODC regression limit. However, the depth profile for the qPCR of prokaryotic 16S rRNA gene copy numbers was more variable and fluctuated around the lower global AODC regression line, with some depths having very low gene copy numbers (< 10^5^ copies cm^−3^; Fig. 3A). At the Flank site (U1316; Fig. 4A), above the mound base, bacterial CARD-FISH detectable cell numbers ranged from 1.1 to 5.8 × 10^5^ cells cm^−3^ with a peak in cell numbers at 51.4 mamb. Below the mound base, CARD-FISH detectable bacterial cell numbers increased to numbers similar to the AODC cell count (mean count of 3.1 × 10^6^ cells cm^−3^) and within the AODC predicted limits. A high DNA copy number of prokaryotic 16S rRNA genes (2.8 × 10^7^ copies cm^−3^) were present near the sediment surface at 54.5 mamb (4 mbsf) and close to the lower AODC global regression limits. Below the sediment surface prokaryotic 16S rRNA gene copy numbers rapidly decreased by 100-fold to their lowest number (2.1 × 10^5^ copies cm^−3^) at the Flank site (at 48.8 mamb) and then remained relatively constant between 5 and 15 × 10^5^ copies cm^−3^ with the exception of a large peak (2.0 × 10^7^ copies cm^−3^) below the mound base at −47.6 mamb which were higher than the AODC counts and near the upper global regression levels.

### Geochemistry and prokaryotic activity

#### Pore water and gas composition

At the Reference site (U1318) sulfate was rapidly removed from seawater concentrations (26.6 mM) at the surface to around 10 mM in the top 14 mbsf and then fluctuated between 10 and 16 mM throughout the hole with two zones of sulfate increase (Fig. 2C). This includes near the erosional surface (84 mbsf) until about 135 mbsf, suggesting oxidation of previously formed metal sulfides. Below this, sulfate concentrations stabilize (∼17 mM) until ∼160 mbsf, where sulfate is again slowly removed until the end of the core (Fig. 2C). Consistent with the high concentrations of sulfate with depth at the Reference site was the absence of methane throughout the hole, reflecting the ability of sulfate-reducing prokaryotes to out-compete methanogens for common substrates (Lovley and Chapelle, 1995). Alkalinity (Fig. 2C, Ferdelman *et al*., 2006) broadly mirrors the sulfate profile, as sulfate removal generates alkalinity. Initially alkalinity increases from 5 to ∼12 mM in the upper part of the core and then rapidly drops to ∼5 mM by 20 mbsf and then gradually increases with depth. Dissolved iron concentrations were high in the near surface (maximum 68 μM) and then stepwise decreased to below detection by 100 mbsf where they remained low until ∼160 mbsf when concentrations increased (maximum 26.7 μM; Fig. 2D). Ferdelman and colleagues (2006) observed that excursions in the profiles of sulfate, alkalinity, dissolved Fe and Mn, and ammonium, corresponded with lithological variations, in particular with erosion surfaces. Pore water acetate concentrations were consistently low, in the range of 1.2– 5.0 μM (Fig. 2D).

In contrast, at the mound sites sulfate was substantially removed. At the Mound site (U1317) sulfate decreased with depth from 27.3 mM at 128.7 mamb (1.4 mbsf) to 2.15 mM below the mound base at −22.21 mamb (168.42 mbsf). When sulfate had reached these low concentrations below the mound base, methane rapidly increased to > 3 mM and generally remained high in deeper layers of the mound base (Fig. 3C). Hence there was a sulfate-methane-transition-zone (SMTZ) and this coincided with a small sulfide peak (0.2 μM) just below the mound base at around −2 mamb (Fig. 3D) suggesting that anaerobic oxidation of methane (AOM) coupled to sulfate reduction was occurring (e.g. Niemann *et al*., 2006). Alkalinity (Fig. 3C) increased with depth to a maximum (∼16 mM) around the mound base and then decreased with depth, reflecting the change from sulfate reduction to methanogenesis. A maximum of dissolved Fe (Fig. 3D; 38 μM) occurred in the near surface (1.4 mbsf; 128.7 mamb), then concentrations dropped rapidly to < 5 μM, with small peaks at −2.6 and −74.69 mamb. Pore water acetate concentrations were again low, in the range of 1.1–4.1 μM above the mound base but increased with depth below the mound base, particularly after about −80 mamb with concentrations reaching 10.3 μM at −122.48 mamb (Fig. 3D).

At the Flank site (U1316), sulfate concentrations (Fig. 4C) stepwise decreased with depth from 25.2 mM at the near surface (56.9 mamb; 1.4 mbsf) to ∼6 mM at −35.6 mamb, where sulfate remained relatively constant until the base of the hole (−86.3 mamb). Low concentrations of methane were only present below the mound base in the Miocene sediments where concentrations began to increase from 0.05 mM at −15.9 mamb (74.2 mbsf) to ∼3.5 mM at the base of the hole and presumably beyond this (Fig. 4C). Alkalinity (Fig. 4C) generally mirrored the sulfate profile increasing with depth from 4.9 mM down to around 10 mM with a peak (13.7 mM) at −23.64 mamb. Similar to the other sites dissolved Fe concentrations were also high near the sediment surface but at concentrations much higher than either the Mound or Reference sites (Fig. 4D; ∼100 μM). Concentrations then stepwise decreased with depth with values much higher above the mound base than below it. Pore water acetate concentrations were very low, in the range of 0.6–8.6 μM with a slight trend of increasing concentrations with depth (Fig. 4D).

#### Rates of prokaryotic activity

Rates of H_2_/CO_2_ and acetate methanogenesis at the Reference site (Fig. 2E) were similar to those at the mound sites (see below), but unlike the two mound sites activity was confined to the upper sediments (> 40 mbsf), which is consistent with the absence of methane at depth at this site. Bacterial activity measured by thymidine incorporation and acetate oxidation (Fig. 2F) was low. Acetate oxidation occurred predominantly above ∼100 mbsf (erosional surface at 84 mbsf) with peaks at 4.25 and 60.9 mbsf (28 and 26 pmol cm^−3^ day^−1^ respectively). Thymidine incorporation was variable in the top 120 mbsf with a peak at 60.9 mbsf (96 pmol cm^−3^ day^−1^), and a steady increase below 120 mbsf reaching 175 pmol cm^−3^ day^−1^ at the end of the core.

At both mound sites (Figs 3E and 4E) low rates of methanogenesis were measured from both H_2_/CO_2_ and acetate (range 0.01–0.75 and 0.00003–0.002 pmol cm^−3^ day^−1^ respectively) above the mound base. Below the mound base only acetate methanogenesis was detected corresponding with increasing methane concentrations (Figs 3C and 4C). Thymidine incorporation and acetate oxidation had a patchy depth distribution throughout the mound sites (Figs 3F and 4F). At the Mound site (U1317) highest rates of thymidine incorporation were measured above the mound base with peaks of 419 and 608 pmol cm^−3^ day^−1^ at 109.85 and 16.89 mamb respectively (Fig. 3F). However, at the Flank site (U1316) thymidine incorporation was the highest below the mound base with peaks at −47.87 mamb (1688 pmol cm^−3^ day^−1^) and at −70.59 mamb (986 pmol cm^−3^ day^−1^) and these corresponded with peaks in acetate oxidation (Fig. 4F). In contrast to the Reference site, both mound sites had maximum rates of acetate oxidation at depth, below the mound base (336 and 185 pmol cm^−3^ day^−1^ at sites U1317 and U1316 respectively).

### Prokaryotic diversity

**Fig. 5:**
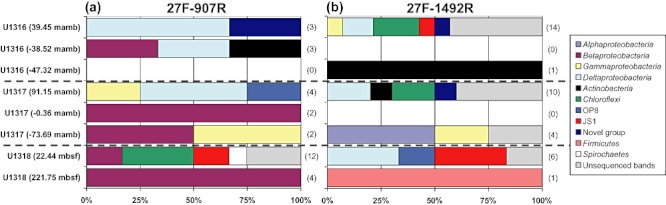
Distribution of bacterial 16S rRNA gene sequences from Challenger Mound sites (U1316 and U1317) and the Reference site (U1318) at different sediment depths using nested PCR-DGGE analysis. a. Nested PCR-DGGE analysis with primer sets 27F-907R and 357F-518R. b. Nested PCR-DGGE analysis with primer sets 27F-1492R and 357F-518R. Numbers of DGGE bands at each depth are shown in parentheses.

At all sites prokaryotic diversity and the numbers of different prokaryotic domains/groups were determined by qPCR of *Bacteria*, *Archaea* and *Geobacteraceae* (metal reducers) 16S rRNA genes, and *dsrA* (sulfate reducers) and *mcrA* (methanogens) genes coupled with PCR-DGGE of bacterial and archaeal 16S rRNA genes and band identification by sequencing (Figs 2–5; Table 1).

**Table 1:** Identity of dominant DGGE bands detected by different nested PCR-DGGE methods in the Porcupine Seabight (IODP Expedition 307)

				Presence^b^ at IODP Expedition 307 site (mamb/mbsf)^c^							
				
PCR-DGGE method^a^	Phylogenetic group	Closest phylotype (accession number)	Sequence similarity (%)	U1316 (39.45)	U1316 (−38.52)	U1316 (−47.32)	U1317 (91.15)	U1317 (−0.36)	U1317 (−73.69)	U1318 (22.44)	U1318 (221.75)
16S rRNA gene bacterial primers 27–907	*Betaproteobacteria*	*Burkholderia* sp. 1HD3 (EF073267)	100	−	+	−	−	−	−	−	−
	*Betaproteobacteria*	*Oxalobacter* sp. HI-D2 (DQ196473)	98	−	−	−	−	+	−	−	−
	*Betaproteobacteria*	*Delftia acidovorans* isolate CI11 (DQ530080)	94	−	−	−	−	+	+	−	−
	*Betaproteobacteria*	Vinyl Chloride enrichment clone PMVC23 (DQ833294)	93	−	−	−	−	−	−	+	−
	*Betaproteobacteria*	Arctic Sea ice clone Elev_16S_794 (EF019643)	94	−	−	−	−	−	−	−	+
	*Betaproteobacteria*	Deep-sea octacoral clone ctg_CGOF251 (DQ395883)	98	−	−	−	−	−	−	•	+
	*Betaproteobacteria*	Deep-well clone S15D-MN15 (AJ583178)	97	−	−	−	−	−	−	−	+
	*Betaproteobacteria*	Mine drainage water clone LOP-7 (DQ241388)	94	−	−	−	−	−	−	−	+
	*Betaproteobacteria*	Contaminated sediment clone 661185 (DQ404909)	97	−	−	−	−	−	−	−	−
	*Gammaproteobacteria*	*Pseudomonas putida* (EF526503)	100	−	−	−	+	−	+	−	−
	*Gammaproteobacteria*	*Bathymodiolus thermophilus* gill symbiont (M99445)	94	−	−	−	−	−	−	−	−
	*Deltaproteobacteria*	Peru margin (ODP Leg 201) clone ODP1230B4.07 (AB177215)	98	+	•	−	−	−	−	−	−
	*Deltaproteobacteria*	Nankai Forearc Basin clone MB-A2-137 (AY093467)	92	•	−	−	•	−	−	−	−
	*Deltaproteobacteria*	Nankai Forearc Basin clone MB-C2-152 (AY093483)	100	−	−	−	+	−	−	−	−
	*Actinobacteria*	Mono Lake clone ML316M-7 (AF447767)	98	−	+	−	−	−	−	+	−
	JS1	Peru margin (ODP Leg 201) clone ODP1230B1.30 (AB177134)	95	−	−	−	−	−	−	+	−
	JS1	Nankai Trough (ODP Leg 190) clone NANK-B7 (AY436531)	100	−	−	−	−	−	−	+	−
	*Chloroflexi*	Urania basin mound clone Urania-2B-30 (AY627589)	100	−	−	−	−	−	−	+	−
	*Chloroflexi*	Peru margin (ODP Leg 201) clone ODP1227B19.06 (AB177057)	100	−	−	−	−	−	−	+	−
	*Chloroflexi*	Peru margin (ODP Leg 201) clone ODP1251B11.21 (AB177310)	99	−	−	−	−	−	−	+	−
	*Chloroflexi*	Peru margin (ODP Leg 201) clone 30-B02 (AJ867602)	97	−	−	−	−	−	−	+	−
	*Spirochaetes*	Microbial mat clone IE053 (AY605139)	92	−	−	−	−	−	−	−	−
	OP8	Peru margin (ODP Leg 201) clone 42-B47 (AJ867599)	97	−	−	−	+	−	−	−	−
	Novel Group	Sea of Okhotsk clone OHKB2.70 (AB094821)	98	+	−	−	−	−	−	−	−
16S rRNA gene bacterial primers 27–1492	*Alphaproteobacteria*	Antarctic freshwater lake clone 60(AM049212)	96	−	−	−	−	−	+	−	−
	*Alphaproteobacteria*	Uncultured *Roseobacter* clone NAC1-1 (AF245614)	99	−	−	−	−	−	+	−	−
	*Gammaproteobacteria*	*Acinetobacter johnsonii* strain WAB1939 (AM184278)	92	+	−	−	−	−	−	−	−
	*Gammaproteobacteria*	Subseafloor clone 33-FL10B99 (AF469278)	100	−	−	−	−	−	+	−	−
	*Deltaproteobacteria*	Nankai Forearc Basin clone MB-A2-137 (AY093467)	92	•	−	−	+	−	−	−	−
	*Deltaproteobacteria*	Peru margin (ODP Leg 201) clone ODP1230B4.07 (AB177215)	94−97	+	−	−	−	−	−	+	−
	*Deltaproteobacteria*	Nankai Forearc Basin clone MB-C2-152 (AY093483)	99	−	−	−	+	−	−	−	−
	*Deltaproteobacteria*	Marine sponge clone 276 (AY485297)	92	−	−	−	−	−	−	+	−
	JS1	Amsterdam mud volcano clone Amsterdam-2B-61 (AY592418)	100	+	−	−	−	−	−	−	−
	JS1	Nankai Trough (ODP Leg 190) clone NANK-B7 (AY436531)	98−100	−	−	−	−	−	−	+	−
	*Chloroflexi*	Ridge flank crustal fluid clone FS274-70B-03 (DQ513102)	98	+	−	−	−	−	−	−	−
	*Chloroflexi*	Sea of Okhotsk clone OHKB2.40 (AB094814)	98	•	−	−	+	−	−	−	−
	*Chloroflexi*	Ridge flank crustal fluid clone FS142-4B-02 (DQ513037)	98	•	−	−	+	−	−	−	−
	*Actinobacteria*	*Brevibacterium samyangensis* strain SST-8 (DQ344485)	96	−	−	+	−	−	−	−	−
	*Actinobacteria*	*Brevibacterium* sp. strain BBH7 (AM158906)	100	−	−	−	+	−	−	−	−
	*Firmicutes*	Salt marsh clone CB_079 (DQ880966)	100	−	−	−	−	−	−	−	+
	OP8	Peru margin (ODP Leg 201) clone 42-B47 (AJ867599)	96	−	−	−	−	−	−	+	−
	Novel Group	Sea of Okhotsk clone OHKB2.70 (AB094821)	98	+	−	−	−	−	−	−	−
	Novel Group	Peru margin (ODP Leg 201) clone ODP1227B18.19 (AB177054)	98	−	−	−	+	−	−	−	−
16S rRNA gene JS1 primers	JS1	Gulf of Mexico clone AT425_EubA5 (AY053496)	93	+	−	−	−	−	−	−	−
	JS1	Nankai Forearc Basin clone MA-A2-104 (AY093459)	99	−	−	−	+	−	−	−	−
	JS1	Cascadia margin (ODP Leg 204) clone ODP1244B5.17	92	−	−	−	+	−	−	−	−
	JS1	Peru margin (ODP Leg 201) clone ODP1230B1.30 (AB177134)	99	−	−	−	−	−	−	+	−
	JS1	Nankai Trough (ODP Leg 190) clone NANK-B7 (AY436531)	99	−	−	−	−	−	−	+	−
	*Chloroflexi*	Sandy carbonate sediment clone CI75cm2.05	97−100	−	−	−				+	
	*Chloroflexi*	Oceanic crust clone FS118-10B-02	100	+	−	−	+	−	−		−
	*Chloroflexi*	Peru margin (ODP Leg 201) clone ODP1227B18.10	95	+	−	−	−	−	−		−
	*Gammaproteobacteria*	Pirite mine drainage *Frateuria* sp. DM-HM	96−97	−	+	−	−	+	+	−	−
	*Gammaproteobacteria*	*Thermomonas hydrothermalis*	100	−	−	−	−	+	−	−	−
	*Deltaproteobacteria*	Sandy carbonate sediment clone CI75cm2.03	90−91	+	−	−	+	−	−	+	−
16S rRNA gene archaeal primers	SAGMEG-1	Peru margin (ODP Leg 201) clone 86-AC3	92	−	−	−	+	−	−	−	−
	SAGMEG-1	Urania basin mound clone Urania-2A-32	97	−	−	−	+	−	−	−	−
	SAGMEG-1	Peru margin (ODP Leg 201) clone 5H2_H23	99−100	+	−	−	+	−	+	−	−
	SAGMEG-1	Urania basin mound clone Urania-2A-16	98	−	−	−	+	−	−	−	−
	SAGMEG-1	Peru margin (ODP Leg 201) clone 1H5_H06	98	+	−	−	−	−	−	−	−
	SAGMEG-1	Peru margin (ODP Leg 201) clone ODP1227A18.12	99	+	−	−	−	−	−	−	−
	SAGMEG-like	Peru margin (ODP Leg 201) clone ODP1227A5.28	98	−	−	−	−	−	+	−	−
	MBG-D	Tidal flat sediment clone BS1-1-79	94−98	−	−	−	−	−	−	+	−
	MBG-D	Nankai Trough (ODP Leg 190) clone NANK-A83	91−93	+	−	−	−	−	−	−	−
	C3	South China Sea clone MD2896-3m.47	96−98	−	−	−	+	−	−	+	−
	MCG	Holocene subsurface sediment clone ITKA-052	91−93	−	−	−	−	−	−	+	−
	MCG	Peru margin (ODP Leg 201) clone 12H3_ar19	98	−	−	−	−	−	−	−	+

a. All bacterial and archaeal PCR products were reamplified by nested PCR prior to DGGE analysis with primers 357F-518R or SAF-PARCH519R respectively.

b. +, identification by sequencing; •, identification by extrapolation of DGGE band position.

c. mamb, metres above mound base (sites U1316 and U1317); mbsf, metres below seafloor (site U1318).

#### Quantification of different prokaryotic groups and functional genes using qPCR

At the Reference site (U1318) the highest numbers of 16S rRNA genes for all prokaryotic groups analyzed were detected in the near surface sediment between 4 and 13.2 mbsf with similar numbers of *Archaea* and *Bacteria* (10^6^−10^7^ copies cm^−3^), which also had similar depth distributions (Fig. 2B). *Archaea* and *Bacteria* decreased steadily with depth down to around 41.7–60.7 mbsf (Fig. 2B), as did the functional genes *mcrA* and *dsrA* and the *Geobacteraceae* 16S rRNA genes. Below 60.7 mbsf gene copy numbers for all prokaryotic groups began to increase with a peak in numbers around the erosional surface at ∼84 mbsf, after which copy numbers of different groups either slowly (*Bacteria*, *dsrA* and *Geobacteraceae*) or rapidly decreased (*Archaea* and *mcrA*). Unlike the mound sites (see below), at the deepest depths of the Reference site (∼220 mbsf and below) archaeal and bacterial 16S rRNA gene numbers were similar. Although archaeal 16S rRNA gene copy numbers increased at depth, no *mcrA* genes were detected below 117.7 mbsf, consistent with the absence of both deep methane and methanogenesis at the Reference site (Fig. 2C and E).

In contrast to the Reference site, the highest numbers of genes detected for all prokaryotic groups at the Mound site (U1317) were in the subsurface sediment around 110.1 mamb (20 mbsf) after which all gene copy numbers decreased to varying extents until 53.1 mamb (Fig. 3B). At ∼17.1–24.1 mamb all prokaryotic groups increased considerably (one to five orders of magnitude). Then gene copy numbers became relatively constant with depth with the exception of *Archaea* 16S rRNA genes which decreased to below detection, except for the deepest sample (−119 mamb). Overall bacterial 16S rRNA gene copy numbers (10^3^−10^6^ copies cm^−3^) were substantially higher than those of *Archaea* (0–10^6^ copies cm^−3^).

At the Flank site (U1316), the highest numbers of genes for all prokaryotic groups were in the near surface sediment at 54.3 mamb (4 mbsf) with a general decrease with depth (Fig. 4B), although subsurface peaks in bacterial (including *Geobacteraceae*) and archaeal 16S rRNA genes were present at 39.3 and −47.6 mamb. *Bacteria* 16S rRNA gene copy numbers were higher than *Archaea* 16S rRNA genes with the exception of the near surface (4 mbsf) and at 10 mamb where copy numbers for the two prokaryotic domains were similar. Copies of the *dsrA* were consistently present (10^2^−10^5^ genes cm^−3^) with the highest numbers in the near surface (to ∼50 mamb) and then declined very slowly with depth. Numbers of *Geobacteraceae* 16S rRNA genes varied between 0 and 10^4^ copies cm^−3^ (Fig. 4B) and with distributions broadly similar to the *dsrA* gene, but with greater decrease with depth. The presence of these genes is consistent with the zones of sulfate removal and presence of dissolved iron throughout most of the sediment at both mound sites. Interestingly, *mcrA* genes were not detected at either of the mound sites, which may suggest that prokaryotes with novel *mcrA* genes are responsible for methanogenesis (Figs 3E and 4E) at the Challenger Mound that are not detected by the primers used in this study.

#### PCR-DGGE analysis of bacterial and archaeal 16S rRNA genes

At the Reference site (U1318) a higher diversity of prokaryotic 16S rRNA gene sequences were identified at 22.44 mbsf than at 221.75 mbsf (Table 1, Fig. 5). For example, at 22.44 mbsf sequences identified by DGGE and band sequencing documented a prokaryotic community comprised of *Betaproteobacteria*, *Deltaproteobacteria*, *Chloroflexi*, *Spirochaetes*, candidate divisions OP8 and JS1, *Euryarchaeota* Marine Benthic Group D (MBG-D; Vetriani *et al.*, 1999), *Crenarcheaota* groups C3 (Inagaki *et al*., 2006) and MCG (Miscellaneous Crenarchaeotic Group; Inagaki *et al*., 2003), whereas at 221.75 mbsf (below the ∼84 mbsf erosional surface) sequences belonged to *Betaproteobacteria*, *Firmicutes* and MCG. Interestingly, in contrast to the mound sites (see below), no *Euryarchaeota* South African Gold Mine Euryarchaeotal Group (SAGMEG; Takai *et al.*, 2001) sequences were detected. Also, many of the phylotypes identified were different from those found at the mound sites (Table 1).

The common bacterial types, based on the combined 16S rRNA gene analysis, present at the two mound sites were *Deltaproteobacteria*, *Gammaproteobacteria*, *Chloroflexi*, candidate division JS1 and a novel bacterial group above the mound base (Table 1; Fig. 5), and *Betaproteobacteria* and *Gammaproteobacteria* below the mound base. In addition, some phyla were only detected at one of the mound sites. For the Mound site (U1317) these were *Actinobacteria* and the candidate division OP8 above the mound base and *Alphaproteobacteria* below the mound, and for the Flank site (U1316) below the base *Deltaproteobacteria* and *Actinobacteria*. Archaeal sequences belonging to the *Euryarchaeota* SAGMEG dominated at both mound sites, although they only occurred above the mound base at the Flank site and were not detected near the mound base at the Mound site. The *Crenarchaeota* group C3 and the *Euryarchaeota* MBG-D group were also detected but only above the mound base at the Mound site and the Flank site respectively (Table 1). In contrast to the Reference site no MCG sequences were detected at the mound sites.

Above the mound base the majority of prokaryotic sequences (Table 1) were related (90–100% sequence similarity) to sequences previously found in other subsurface habitats like subseafloor and gas hydrate sediments (Nankai Trough, Peru Margin, Sea of Okhotsk, Cascadia Margin, South China Sea), crustal fluids (Juan de Fuca Ridge), sandy carbonate sediments (Kaneohe Bay) and carbonate mounds (Urania Basin). Whereas below the mound base, bacterial sequences found were related (94–100% sequence similarity) to cultured bacterial genera such as *Frateuria*, *Burkholderia*, *Oxalobacter* and *Delftia*. In addition, at the Mound site (U1317) some bacterial sequences found below the mound base were related (93–100% sequence similarity) to sequences from other environments such as river sediment (vinyl chloride enrichment), Antarctic fresh water lake, North Atlantic seawater, hydrothermal vent fluids (Axial Volcano, Juan de Fuca Ridge), although all archaeal sequences found here were related (98–100% sequence similarity) to sequences previously found in subsurface sediments (Peru Margin, Table 1).

#### Culturable prokaryotic diversity

At the Reference (U1318) and Flank (U1316) sites, culturable bacteria (fermentative heterotrophs, metal reducers, sulfate reducers and acetogens) were only detected below the erosional surface (∼84 mbsf) or below the mound base (from −38.85 mamb; 90.35 mbsf) respectively (Figs 2G and 4G). In contrast, culturable bacteria at the Mound site (U1317) were present at above and below the mound base; however, acetogens were only found above the mound base (Fig. 3G). At the Mound site viable cell numbers, when detected, ranged from 40 to 800 cells cm^−3^ except for at sediment depth 16.89 mamb where both heterotrophic and sulfate-reducing bacteria were higher and at their maxima (21 200 and 1700 cells cm^−3^ respectively). Peaks in all types of culturable cell numbers occurred at the Flank site at −47.87 mamb, and this corresponded with a peak in prokaryotic 16S rRNA genes and high bacterial CARD-FISH counts (Fig. 4A). At the Reference site, viable cell numbers ranged from ∼200 to 600 cells cm^−3^ except for heterotrophic bacteria at 153.1 mbsf which were 1640 cells cm^−3^, with sulfate reducers and acetogens alone being detected at 221.1 mbsf (Fig. 2G). Methanogens were not enriched in any of the samples at any of three sites.

### Comparisons and correlations

On average at all three sites the percentages of the following relative to AODC counts were: bacterial CARD-FISH (13%), 16S rRNA gene copy numbers for prokaryotes (37%), *Bacteria* (17%), *Archaea* (14%), *Geobacteraceae* (0.01%), and the functional genes *dsrA* (0.07%) and *mcrA* (2.4%). Hence, bacterial and archaeal 16S rRNA gene copy numbers are similar and *Bacteria* or functional genes involved in terminal oxidation reactions represent only a small proportion of the total population. Interestingly, the combined total of bacterial and archaeal 16S rRNA gene copy numbers as a percentage of the AODC (31%) was similar to that for prokaryotes. The sites with the highest percentage of these cell-based measurements relative to the AODC cell counts were as follows: for bacterial CARD-FISH the mound (22%); for prokaryotic 16S rRNA genes the Flank and Reference sites (42% and 45% respectively); for bacterial and archaeal 16S rRNA genes the Flank site (22% and 23% respectively); for *Geobacteraceae* 16S rRNA genes the Reference site (0.02%); for *dsrA* genes the Flank site (0.11%); and for *mcrA* genes the Reference site (7.1%). The Reference site was the only site where *mcrA* genes were detected, and surprisingly it was the only site without measurable methane (Fig. 2). Reasonable rates of methanogenesis, however, were detected, but like the *mcrA* gene, methanogenic activity was absent from the deeper part of the core (not detected below 120 and 40 mbsf respectively). In contrast, the *dsrA* gene was present throughout each of the three sites and its presence is consistent with sulfate removal at all sites (Figs 2–4). However, the depth distribution of *dsrA* genes tended to be similar for all three sites (the highest in near surface sediment and then slowly decreasing with depth) despite these sites having very different depth profiles of sulfate removal.

There are several significant correlations (*P* < 0.05 to < 0.002) between the different measures of the cellular prokaryotic community composition at the sites. Data combined from all three sites shows highly significant correlations particularly (*r* > 0.7, *n* = 30) between 16S rRNA gene copies for prokaryotes and bacteria, 16S rRNA gene copies for *Bacteria*, *Archaea* and *Geobacteraceae* and the *dsrA* gene copy number. Many relationships with bacterial CARD-FISH were negative and this was also observed at individual sites. At the two mound sites there was a strong (*r* > 0.9) correlation between total prokaryotic 16S rRNA gene copy number and those for both *Bacteria* and *Archaea*, and in samples above and below the mound base. In the Reference site, however, the correlation between 16S rRNA gene copies for prokaryotes and those for *Archaea* was not significant.

## Discussion

The Challenger Mound in the Belgica Mound Province of the Porcupine Seabight is the first mound structure to be successfully cored deeper than 12 m (Ferdelman *et al*., 2006), and was drilled by IODP Expedition 307 to achieve several objectives, including to establish whether the mound was rooted on a carbonate hardground (lithified surface) produced by microbial utilization of seep hydrocarbons as proposed by Hovland and colleagues (1994). However, it now seems unlikely that this was the mode of formation, as the mound base was not carbonate hardground but instead was Miocene (from ∼5 Mya) firmground (unlithified firm surface) produced by erosional processes (Ferdelman *et al*., 2006) and which occurred at all three sites. Drilling revealed that the Challenger Mound is composed of unlithified coral-bearing sediments (floatstone, rudstone and wackestone; Fig. 1) almost entirely from dead *L. pertusa*. Hence, it is probable that the mound was formed entirely by the framework building and sediment baffling capacity of cold-water corals (Wheeler *et al*., 2007) after their attachment to the erosional surface. However, despite this, there were significant prokaryotic populations and activities at the two Challenger Mound sites, including above and below the mound base, and considerable microbiological differences between these mound sites and a nearby non-mound Reference site.

Both the Flank (U1316) and Challenger Mound (U1317) sites contained significant numbers of subsurface prokaryotic cells. However, total cell numbers (AODC) at some depth zones were lower than the global average prokaryotic cell numbers for other subseafloor sediments (Parkes *et al*., 2000), including those present at the Reference site (U1318). Despite these low cell numbers, prokaryotic activity in the deeper subsurface sediments of the mound sites were higher than that measured in the Reference site (more sulfate removed, higher acetate oxidation and thymidine incorporation, presence of deep acetoclastic methanogenesis), and this activity difference was also consistent with the formation of methane beneath the mound base (Figs 3 and 4). In addition, the presence of significant thymidine incorporation and acetate oxidation below the mound base indicates the presence of active heterotrophic bacterial populations which is consistent with the culturable bacteria identified by Most Probable Number (MPN) (Figs 3 and 4). Gas wetness and isotope data suggest that methane at the mound sites is a mixture of biogenic and thermogenic origin and that both have migrated from a deeper source (Mangelsdorf *et al*., 2008). This would appear to be consistent with the suggestion of Hovland and colleagues (1994) that, ‘The seepage of hydrocarbons is suspected to have caused local eutrophication or “fertilization” by providing nutrients to bacteria, which in turn are part of the food chain for higher organisms’, including cold-water corals. However as sulfate-reducing prokaryotes out-compete methanogens for competitive substrates (Capone and Kiene, 1988), the presence of methane only when there has been a significant reduction in sulfate concentrations at the mound sites (to 3–5 mM) suggests that methane is being actively produced by prokaryotes at these sites, and this is consistent with measurable methanogenic activity, albeit at low rates (Figs 3 and 4).

Relatedly, there is a SMTZ at both mound sites: just below the mound base at the Mound site (Fig. 3) and deeper than the mound base at the Flank site (below about −40 mamb; Fig. 4). At other locations a SMTZ is associated with AOM (e.g. Peru Margin, Parkes *et al*., 2005; Hydrate Ridge, Treude *et al*., 2003; Eckernförde Bay, Treude *et al*., 2005; Gulf of Cadiz, Niemann *et al*., 2006). Although, the potentially responsible *Archaea* (ANME) sequences (Boetius *et al*., 2000) were not detected in this study (Table 1), this is often the case with deep sediments (Parkes *et al*., 2005; Inagaki *et al*., 2006); hence, the presence of a SMTZ near or below the mound base indicates that there is currently some AOM activity within the mound base. However rates are probably low, considering the shallow depth gradients of both sulfate and methane (Figs 3 and 4), which is consistent with absence of significant carbonate hardground (Ferdelman *et al*., 2006). It therefore seems reasonable that the presence of subsurface methanogenesis at the mound sites is a consequence of mound formation stimulating prokaryotic activity [e.g. biodegradable organic matter production/trapping leading to enhanced sulfate removal, and hence, methanogenesis; higher acetate concentrations (maximum acetate mound sites ∼10 μM compared with ∼5 μM for Reference site)] rather than a cause of mound formation. However, this does not exclude the potential for microseepage providing additional methane from depth, which also would not lead to carbonate hardground formation (Hovland, 2008).

As found in other subseafloor sediments (D'Hondt *et al*., 2004; Parkes *et al*., 2005) prokaryotic activity at IODP Expedition 307 sites is quite diverse and activities do not follow the expected depth distributions based on a sequence of reactions providing decreasing energy yield (Telling *et al*., 2004), as the theoretically competitive activities of metal reduction, sulfate reduction and methanogenesis often co-occur at the same depths (Figs 3 and 4). The reasons for the co-occurrence of competitive prokaryotic activities in subsurface sediments is unclear (D'Hondt *et al*., 2004), but it may be related to these low energy flux environments not resulting in any one metabolic group of prokaryotes being able to become dominant. Both the 16S rRNA gene and cultivation (heterotrophs, metal and sulfate reducers and acetogens) diversity data support the presence of a diverse prokaryotic community. In particular, the qPCR data show the co-occurrence of the metal-reducing *Geobacteraceae*, the sulfate-reducing specific functional *dsrA* gene (Figs 3 and 4) and at the Reference site also the methanogen-specific functional *mcrA* gene (Fig. 2). However, these data have to be interpreted with some caution, as it is surprising that the *mcrA* gene was not detected at the Challenger Mound sites despite the presence of both deep methane gas and active methanogenesis (Figs 3 and 4), yet this gene was detected at the Reference site without detectable methane gas or associated deep methanogenesis (Fig. 2). In addition, there was also not a close correspondence between the depth distribution of the *dsrA* functional gene and zones and intensity of sulfate removal [e.g. Mound site (U1317) had almost complete sulfate removal at depth while the Reference site had limited sulfate removal, yet average *dsrA* gene copies at the Mound site was 9.16 × 10^2^ copies cm^−3^ compared with 7.56 × 10^3^ copies cm^−3^ for the Reference site]. However, we know little about the real depth distribution of different metabolic prokaryotic groups and how this relates to specific activities, as terminal oxidizers can be part of syntrophic consortia with different metabolic reactions (Stams *et al*., 2006) and are rarely detected in phylogenetic surveys, as occurred at these sites (Table 1). Overall, 16S rRNA genes of *Geobacteraceae*, and *dsrA* and *mcrA* genes represented only a small proportion of the total AODC cell count (means for all data, 0.01%, 0.07% and 2.37% respectively) which probably explains why they were not detected by the PCR-DGGE analysis.

CARD-FISH ‘active’ bacterial cell counts represented on average a higher percentage of the AODC total count at the two mound sites (Flank and Mound 11.6% and 21.9% respectively) than at the Reference site (5.0%). This is consistent with the overall higher measured activities and numbers of culturable prokaryotes in the mound sites. However, the proportion of total ‘active’ cells present must be higher than these estimates, as archaeal cells were not detected by CARD-FISH, but *Archaea* were present, as shown by qPCR (Figs 2–4), PCR-DGGE (Table 1) and indirectly by the presence of active methanogenesis at the mound sites. CARD-FISH counts of archaeal cell numbers may have been below detection due to sequence mismatches of the archaeal probe 915 with sequences from some archaeal groups often found in gene libraries of deeply buried sediments (Teske and Sørensen, 2008). In addition, the considerable number of negative correlations between bacterial CARD-FISH and other measures of cell abundance also suggests that CARD-FISH in this study is not detecting the complete prokaryotic population.

Based on the qPCR data *Bacteria* and *Archaea*, respectively, represent the following percentages of the detected prokaryotic 16S rRNA genes: 52% and 54% for the Flank site, 59% and 13% for the Mound site and 39% and 35% for the Reference site. Hence, there is considerable variability in the proportions of *Bacteria* and *Archaea* in these three geographically close sites. This may be partly explained by primer sequence mismatches (Baker *et al*., 2003; Teske and Sørensen, 2008), as previously described, together with the differences in archaeal diversity at each site (Table 1). Previous qPCR results for subsurface sediments from the Peru continental margin (Schippers *et al*., 2005; Schippers and Neretin, 2006) showed that *Archaea* 16S rRNA genes were one to three orders of magnitude lower than those for *Bacteria*, and hence, differ considerably from the Porcupine Seabight deep sediments studied here. Taking into consideration that three different primers were used for bacterial 16S rRNA gene amplification compared with one archaeal primer, the qualitative PCR-DGGE results (Table 1) also suggest similar proportions of bacterial and archaeal sequences. This has also recently been found for north-east Pacific ridge-flank sediments (Engelen *et al*., 2008). However, in none of the molecular analyses were *Archaea* shown to be dominant, as reported in some subseafloor sediment studies (Biddle *et al*., 2006; Lipp *et al*., 2008). Furthermore, there was an ∼100 m zone beneath the mound base at the Mound site where archaeal 16S rRNA gene copies were not detected (Fig. 3) and below the mound base at the Flank site archaeal 16S rRNA gene copy numbers were variably low (Fig. 4). Hence, these data also demonstrate considerable variability between the proportions of *Bacteria* and *Archaea* with depth at Porcupine Seabight sites.

Surprisingly, culturable prokaryotes (MPN) appeared to be absent in the top ∼80 mbsf at both the Reference and Flank sites (Figs 2 and 4). This is despite considerable numbers of culturable prokaryotes occurring in deeper layers at these sites (e.g. maximum ∼70 000 SRB cm^−3^ at Flank site) and the presence of culturable prokaryotes at much shallower depths in the Mound site (Fig. 3). Enrichments conducted in parallel to the MPN series were also negative for growth in the upper zone of the Flank and Reference sites and the sediment inoculum remained undispersed after several months of incubation and repeated shaking. The zones with unculturable prokaryotes at both sites broadly correspond to the presence of silty-clay layers containing considerable amounts of highly reworked sediment (Ferdelman *et al*., 2006). The undispersible features of these sediments could also have prevented effective dilution, inoculation and growth in the MPN plates, resulting in the apparent absence of culturable cells. Nevertheless, cells *in situ* were viable throughout the cores at each site, as demonstrated by the presence of: (i) cells containing ribosomes detected by CARD-FISH, (ii) significant copy numbers of 16S rRNA and functional genes, and (iii) radiotracer and geochemical measurements of activity (heterotrophic activity and growth, iron and sulfate reduction, methanogenesis). These results are consistent with the presence of a number of different viable prokaryotic groups (heterotrophs, metal and sulfate reducers, and acetogens) when cultivation was successful.

Consistent with the presence of diverse culturable prokaryotes and metabolic activities, a range of different types of *Bacteria* and *Archaea* were detected by cultivation-independent approaches (Table 1, Fig. 5), several of which are common in other subseafloor sediments (*Gammaproteobacteria*, *Chloroflexi*, JS1, SAGMEG, MBG-D and MCG; Webster *et al*., 2004; 2006; Inagaki *et al*., 2006; Teske, 2006; Fry *et al*., 2008). All of the *Archaea* and many of the *Bacteria* are related to uncultured environmental sequences, and consistent with this no *Archaea* were isolated; however, the *Gammaproteobacteria* genera, *Vibrio* and *Shewanella*, were common among the bacterial isolates obtained (F. Mathes, G. Webster, R.J. Parkes, A.J. Weightman, H. Sass, unpubl. results). Interestingly, no SAGMEG sequences were present in the Reference site and no MCG sequences were present in the mound sites. MCG sequences are often the dominant *Archaea* in other deep subseafloor sediments (e.g. Peru Margin Sediments, Parkes *et al*., 2005; Sea of Okhotsk ash layers, Inagaki *et al*., 2003; Fry *et al*., 2008). However, it is possible that some MCG phylotypes may have been missed due to sequence mismatches with the archaeal 16S rRNA gene primers used for DGGE analysis (Webster *et al*., 2006; Teske and Sørensen, 2008). In addition, a novel bacterial group identified at both mound sites in samples above the mound base had only previously been detected in subsurface sediments of the Peru Margin (Inagaki *et al*., 2006) and the Sea of Okhotsk (Inagaki *et al*., 2003); interestingly, these regions have seeps (Judd and Hovland, 2007), and hence, are distinct from other subsurface sediments but not necessarily from carbonate mounds.

Overall, fewer prokaryotic sequences were detected at depth at all sites (Table 1, Fig. 5) despite some activities being elevated in deeper layers (Figs 2–4). However, the majority of these sequences were mainly related to uncultured groups of prokaryotes from a range of different environments, and therefore it is unclear what metabolisms are responsible for the deep elevated thymidine incorporation or acetate oxidation, particularly at the mound sites, and in the apparent absence of significant iron and sulfate reduction.

These data demonstrate that there is a significant and active prokaryotic population in the subsurface of the Challenger Mound, both within and beneath the carbonate mound. Although total cell numbers at certain depths are lower than the global average for other subseafloor sediments, and prokaryotic activities of iron reduction, sulfate removal, methanogenesis and acetate oxidation are relatively low, they are more intense than in the Reference site. This suggests that the presence of the mound has enhanced prokaryotic activity. In addition, there is some stimulation of prokaryotic activity in the deepest Miocene (> 10 Ma) sediments and the potential for AOM activity below the mound base. Significant numbers of both bacterial and archaeal cells are present, with neither overall dominant, and these are related to sequences commonly found in other subseafloor sediments. With an estimate of some 1600 mounds in the Porcupine Basin alone (Ferdelman *et al*., 2006), carbonate mound systems may represent a significant subseafloor prokaryotic habitat.

## Experimental procedures

### Sample collection

Samples were collected in May 2005 by shipboard scientists on the JOIDES Resolution during the IODP Expedition 307. Sediment cores were taken from the Flank site U1316 (51°22.56′N, 11°43.81′W; 965 m water depth) located in down-slope sediment deposits ∼700 m south-west of the Challenger Mound; Mound site U1317 located on the north-west shoulder of the Challenger Mound (51°22.8′N, 11°43.1′W; 781–815 m water depth); and a Reference site U1318 (51°26.16′N, 11°33.0′W; 423 m water depth) located on the eastern slope of the Porcupine Seabight. All cores for microbiological analysis were drilled with full microbiological contamination checks (Ferdelman *et al*., 2006) and subsequent 10–20 cm whole round cores (WRC) were aseptically cut on board ship (Parkes *et al*., 1995), capped and stored. For molecular analysis the 10 cm WRC were immediately frozen, transported and stored at −80°C until processed. Samples for activity measurements were taken from adjacent 20 cm WRC and stored and transported at 4°C, to Cardiff University for analysis. Due to logistical constraints subsampling and further processing of the 4°C samples occurred ∼8 weeks after collection, this is a typical storage period incurred in IODP microbiological studies (e.g. Wellsbury *et al*., 2002).

Drilling constraints during IODP Expedition 307 due to differences in sediment lithology, led to the need for multiple holes to be drilled which had different depths to the mound base (Ferdelman *et al*., 2006). For ease of interpretation data from different holes at the Challenger Mound sites U1316 and U1317 are expressed as metres above mound base (mamb). Reference sediment site U1318 sample depths are expressed as metres below the seafloor (mbsf).

### Geochemistry

Pore water samples were measured for volatile fatty acids, sulfate and chloride on a Dionex ICS-2000 Ion Chromatography System equipped with an AS50 autosampler (Dionex UK). Chromatographic separation was conducted on two Ionpac AS15 columns in series and the determination of species was carried out using an Anion Self-Regenerating Suppressor (ASRS-ULTRA II 4-mm) unit in combination with a DS6 heated conductivity cell. The gradient programme was as followed: 6 mM KOH (38 min), 16 mM KOH min^−1^ to 70 mM (17 min), 64 mM KOH min^−1^ to 6 mM (12 min).

### AODC and CARD-FISH

Samples for AODC and CARD-FISH were fixed on board ship immediately after sampling in 2% and 3% (v/v) formaldehyde respectively. CARD-FISH samples were then washed with phosphate-buffered saline (PBS) and stored in ethanol : PBS (1:1, v : v) at −20°C for post-cruise analyses. AODC was based on that described by Fry (1988). Between 5 and 50 μl of a formaldehyde-preserved subsample was stained [0.1% (w/v) acridine orange] and then counted on a black polycarbonate membrane filter (0.2 μm) under an epifluorescence microscope. CARD-FISH was carried out as described (Pernthaler *et al*., 2002; Schippers *et al*., 2005) and filters were hybridized for *Archaea* and *Bacteria* using probes ARCH915 or EUB338-I to -III as a mixture (Table 2).

**Table 2:** PCR primers and probes used in this study

Primer/probe	Target gene	Sequence^a^ (5′−3′)	Reference	Approach
27F	*Bacteria* 16S rRNA	AGA GTT TGA TCM TGG CTC AG	Lane (1991)	PCR-DGGE
1492R	*Bacteria* 16S rRNA	GGT TAC CTT GTT ACG ACT T	Lane (1991)	PCR-DGGE
907R	*Bacteria* 16S rRNA	CCG TCA ATT CMT TTG AGT TT	Muyzer *et al*. (1998)	PCR-DGGE
63F	*Bacteria* 16S rRNA	CAG GCC TAA CAC ATG CAA GTC	Marchesi *et al*. (1998)	PCR-DGGE
665R	JS1 candidate division 16S rRNA	ACC GGG AAT TCC ACY TYC CT	Webster *et al*. (2004)	PCR-DGGE
357F^b^	*Bacteria* 16S rRNA	CCT ACG GGA GGC AGC AG	Muyzer *et al*. (1993)	PCR-DGGE
518R	Universal 16S rRNA	ATT ACC GCG GCT GCT GG	Muyzer *et al*. (1993)	PCR-DGGE
109F	*Archaea* 16S rRNA	ACK GCT CAG TAA CAC GT	Grosskopf *et al*. (1998)	PCR-DGGE
958R	*Archaea* 16S rRNA	YCC GGC GTT GAM TCC AAT T	DeLong (1992)	PCR-DGGE
SAF^b^	*Archaea* 16S rRNA	CCT AYG GGG CGC AGM RGG	Nicol *et al*. (2003)	PCR-DGGE
PARCH519R	*Archaea* 16S rRNA	TTA CCG CGG CKG CTG	Øvreas *et al*. (1997)	PCR-DGGE
EUB338	*Bacteria* 16S rRNA	GCT GCC TCC CGT AGG AGT	Amann *et al*. (1990)	CARD-FISH
EUB338-II	*Bacteria* 16S rRNA	GCA GCC ACC CGT AGG TGT	Daims *et al*. (1999)	CARD-FISH
EUB338-III	*Bacteria* 16S rRNA	GCT GCC ACC CGT AGG TGT	Daims *et al*. (1999)	CARD-FISH
ARCH915	*Archaea* 16S rRNA	GTG CTC CCC CGC CAA TTC CT	Stahl and Amann (1991)	CARD-FISH
Uni340F	Universal 16S rRNA	CCT ACG GGR BGC ASC AG	Takai and Horikoshi (2000)	TaqMan qPCR
Uni806R	Universal 16S rRNA	GGA CTA CNN GGG TAT CTA AT	Takai and Horikoshi (2000)	TaqMan qPCR
Uni516F	Universal 16S rRNA	TGY CAG CMG CCG CGG TAA HAC VNR S	Takai and Horikoshi (2000)	TaqMan qPCR
331F	*Bacteria* 16S rRNA	TCC TAC GGG AGG CAG CAG T	Nadkarni *et al*. (2002)	TaqMan qPCR
797R	*Bacteria* 16S rRNA	GGA CTA CCA GGG TAT CTA ATC CTG TT	Nadkarni *et al*. (2002)	TaqMan qPCR
Nadkarni UNI	*Bacteria* 16S rRNA	CGT ATT ACC GCG GCT GCT GGC AC	Nadkarni *et al*. (2002)	TaqMan qPCR
Arch349F	*Archaea* 16S rRNA	GYG CAS CAG KCG MGA AW	Takai and Horikoshi (2000)	TaqMan qPCR
Arch806R	*Archaea* 16S rRNA	GGA CTA CVS GGG TAT CTA AT	Takai and Horikoshi (2000)	TaqMan qPCR
Arch516F	*Archaea* 16S rRNA	TGY CAG CCG CCG CGG TAA HAC CVG C	Takai and Horikoshi (2000)	TaqMan qPCR
GEO494F	*Geobacteraceae* 16S rRNA	AGG AAG CAC CGG CTA ACT CC	Holmes *et al*. (2002)	SYBR Green qPCR
GEO825R	*Geobacteraceae* 16S rRNA	TAC CCG CRA CAC CTA GT	Anderson *et al*. (1998)	SYBR Green qPCR
ME1f	*mcrA*	GCM ATG CAR ATH GGW ATG TC	Hales *et al*. (1996)	SYBR Green qPCR
ME3r	*mcrA*	TGT GTG AAS CCK ACD CCA CC	Wilms *et al*. (2007)	SYBR Green qPCR
DSR1F+	*dsrA*	ACS CAC TGG AAG CAC GGC GG	Kondo *et al*. (2004)	SYBR Green qPCR
DSR-R	*dsrA*	GTG GMR CCG TGC AKRTTG G	Kondo *et al*. (2004)	SYBR Green qPCR

a. B = G, T or C; D = G, A or T; H = A, T or C; K = G or T; M = A or C; N = G, A, T or C; R = A or G; S = G or C; V = G, A or C; W = A or T; Y = C or T.

b. For DGGE this primer has the GC-clamp at the 5′ end, CGCCCGCCGCGCGCGGCGGGCGGGGCGGGGGCACGGGGGG (Muyzer *et al*., 1993).

### Thymidine incorporation into DNA, acetate oxidation and rates of methanogenesis

Samples for activity measurements were taken from the centre of WRC with sterile 5 ml syringes (luer end removed) under anaerobic and aseptic handling conditions (Parkes *et al*., 1995) and sealed with sterile, black butyl-rubber Subaseals (William Freeman). For more compacted deeper samples (site U1318) or samples below the mound base (sites U1316 and U1317), WRC were handled in an anaerobic chamber; sediment adjacent to the core liner was aseptically removed and the core broken into pieces and ground into a coarse powder. Crushed sediment samples (5 cm^3^) plus sterile, anaerobic artificial seawater (ASW) (5 ml) were sealed into 50 ml volume serum bottles. All samples were then equilibrated anaerobically at 10°C for approximately 1 day prior to further processing. Prokaryotic activity was measured by injecting the sediment samples individually with ^3^H-thymidine (38 μl, 0.81 MBq), ^14^C-acetic acid (sodium salt, 7.6 μl, 0.05 MBq) and ^14^C-bicarbonate (7.4 μl, 0.13 MBq), for estimating heterotrophic growth, acetoclastic methanogenesis, acetate oxidation and hydrogenotrophic methanogenesis, respectively, as described (Parkes *et al*., 2005). Ten syringe mini-cores or crushed core samples were used at each depth for each isotope, with one frozen immediately after injection as a blank and any activity was subtracted from the incubated samples. For mini-cores isotope was injected laterally along the centre-line of the syringe using an injection rig (Parkes *et al*., 1995). Injected samples were incubated, under N_2_ for mini-cores, at 10°C (close to the average down-core temperature) in triplicate for three different incubation times, with longer incubations for deeper depths (thymidine from 4 h to 4 days, acetate from 6 h to 7 days, bicarbonate from 2 to 35 days). Incubations were terminated and stored by freezing at −20°C.

### DNA extraction

Potential drilling contamination was restricted during molecular analysis by subsampling from the WRC centre in the laboratory under aseptic conditions (Webster *et al*., 2003). Total community genomic DNA was extracted from all sediment samples using the FastDNA Spin Kit for Soil (MP Biomedicals) with modification as essentially described in Webster and colleagues (2003). The exception was that the 200 μg of polyadenylic acid (polyA; Sigma or Roche Diagnostics) added to the sediment/lysis buffer mix prior to cell lysis was pre-treated with DNase I (Promega; 37°C for 30 min followed by 75°C for 15 min) to minimize contamination by exogenous DNA within the polyA preparation. All DNA extracts were aliquoted and stored at −80°C until required for PCR amplification. Additionally, a negative control DNA extraction (where no sediment was added to the extraction reagents) was also carried out and analyzed.

### PCR-DGGE analysis

Prokaryotic 16S rRNA genes were amplified by PCR from sediment DNA extracts using the general bacterial primer combinations 27F-907R and 27F-1492R, the JS1-targeted primers 63F-665R and the archaeal primers 109F-958R as previously described (Table 2; Webster *et al*., 2004; 2006). All bacterial and archaeal 16S rRNA gene PCR products were then re-amplified by nested PCR with primers 357FGC-518R or SAF-PARCH519R as described (Table 2; Webster *et al*., 2003; 2006).

DGGE was then carried out as described by Webster and colleagues (2003). PCR products (approximately 100 ng of each PCR product) were separated using a DCode Universal Mutation Detection System (Bio-Rad Laboratories) and 1-mm-thick (16 × 16 cm glass plates) 8% (w/v) polyacrylamide gels with a gradient of denaturant between 30% and 60%. Gels were poured with a 50 ml volume Gradient Mixer (Fisher Scientific) and prepared with 1× TAE buffer. Electrophoresis was carried out at 200 V for 5 h (with an initial 10 min at 80 V) at 60°C in 1× TAE buffer. Polyacrylamide gels were stained with SYBRGold nucleic acid gel stain (Molecular Probes) for 30 min and viewed under UV. Gel images were captured with a Gene Genius Bio Imaging System (Syngene).

### qPCR analysis

Real-time qPCR was used to determine the copy number of the 16S rRNA genes of prokaryotes (universal primer set), *Archaea*, *Bacteria*, and the Fe(III)- and Mn(IV)-reducing family *Geobacteraceae* and the functional genes disimilatory sulfite reductase (*dsrA*) and methyl coenzyme M reductase (*mcrA*). All qPCR methods, primers and probes are listed in Table 2 and all methods were as described in Schippers and Neretin (2006) and Wilms and colleagues (2007).

### Enumeration of culturable prokaryotes

#### Growth media

Sediment slurries and MPN series were prepared using a bicarbonate-buffered ASW medium containing (g l^−1^): NaCl (24.3), MgCl_2_·6H_2_O (10), CaCl_2_·H_2_O (1.5), KCl (0.66), Na_2_SO_4_ (4), KBr (0.1), H_3_BO_3_ (0.025), SrCl_2_·6H_2_O (0.04), NH_4_Cl (0.021), KH_2_PO_4_ (0.0054), NaF (0.003). The ASW medium was supplemented with 1 ml l^−1^ of unchelated trace element solution SL10 and 0.2 ml l^−1^ of a selenite and tungstate solution (Widdel and Bak, 1992). After autoclaving, the medium was cooled under N_2_/CO_2_ (80/20, v/v) and 30 ml l^−1^ of a 1 M NaHCO_3_ solution was added from sterile stocks. The medium was reduced by adding sterile Na_2_S and acid FeCl_2_ solutions to final concentrations of 1.5 mmol l^−1^ and 0.5 mmol l^−1^ respectively. The pH of the reduced medium was adjusted to 7.2–7.4 with sterile HCl or Na_2_CO_3_ if necessary. Media used for MPN series were amended with 10 ml l^−1^ of a vitamins solution (Balch *et al*., 1979).

#### Preparation of MPN series

Five different substrate combinations were used for MPN series targeting different physiological groups. (i) Medium for heterotrophic bacteria (fermenters) contained 0.1 mmol l^−1^ of each of the 20 common l-amino acids; the short-chain fatty acids formate, acetate, propionate, butyrate, valerate, caproate; the alcohols methanol, ethanol, *n*-propanol, *n*-butanol; and dl-malate, fumarate, succinate, dl-lactate, glycerol, glucose (Köpke *et al*., 2005) and 0.5 mmol l^−1^ sodium nitrate. (ii) Medium for metal-reducing bacteria contained 3 mmol l^−1^ sodium lactate, 5 mmol l^−1^ sodium acetate, 2 mmol l^−1^ sodium formate, 2 mmol l^−1^ sodium propionate, 1 mmol l^−1^ sodium butyrate with 20 mmol l^−1^ amorphous iron hydroxide and 10 mmol l^−1^ manganese oxides (Köpke *et al*., 2005) as electron acceptors. (iii) Medium for sulfate-reducing bacteria contained the same electron donors as the medium for metal reducers, but had 10 mmol l^−1^ sodium sulfate as an electron acceptor instead of metal oxides. (iv) Medium for methanogens contained 2 mmol l^−1^ sodium formate and 4 mmol l^−1^ sodium acetate. (v) Medium for acetogens did not contain any additional organic compounds. Media for methanogens and acetogens had a H_2_/CO_2_ headspace.

Sediment slurries used for inoculation of MPN series were made by suspending 2 cm^3^ of sediment in 18 ml of substrate-free ASW medium. The MPN series were prepared in polypropylene 96-deep-well plates (Beckman Coulter) and set up in an anaerobic cabinet (Süß*et al*., 2004; Köpke *et al*., 2005). Each plate contained four different MPN series with three replicates and six 10-fold dilutions. Furthermore, each plate contained four uninoculated dilution series as control. The inoculated and sealed MPN plates were placed into gas-tight plastic bags with a gas generating catalyst system for anoxic conditions (Anaerocult A mini, Merck). Most Probable Number series for heterotrophs, metal and sulfate reducers were incubated for 6–8 months, and those for acetogens and methanogens for at least 12 months and at 10°C.

#### Analysis of MPN series

Gas-tight plastic bags containing MPN plates were transferred into an anaerobic chamber. Headspace samples were taken from the plates containing media for methanogens and acetogens and analyzed for the presence of methane by gas chromatography (Arnel Modified Model 2101 Natural Gas Analyser, Perkin Elmer). A 100 μl of sample from each well was transferred into a black 96-well plate with 25 μl of SYBR Green I (Molecular Probes) working solution (stock diluted 1: 2000 in TE buffer; Martens-Habbena and Sass, 2006). Plates were incubated for 6 h in the dark before analysis on a fluorescence multiplate reader (485 nm excitation, 520 nm emission; FluoroCount Microplate Flurometer, Packard). Growth was scored as positive if fluorescence was at least twice as high as the average fluorescence in uninoculated controls. Most Probable Number counts and confidence levels were calculated as described (Klee, 1993).

### Statistical analysis

Comparisons between the estimates of numbers of different prokaryotic types and cell numbers were obtained using Pearson's Correlation on the log-normalized data.
